# Draft genomes of four enterotoxigenic *Escherichia coli* (ETEC) clinical isolates from China and Bangladesh

**DOI:** 10.1186/s13099-015-0059-z

**Published:** 2015-04-08

**Authors:** Fei Liu, Xi Yang, Zhiyun Wang, Matilda Nicklasson, Firdausi Qadri, Yong Yi, Yuying Zhu, Na Lv, Jing Li, Ruifen Zhang, Huijuan Guo, Baoli Zhu, Åsa Sjöling, Yongfei Hu

**Affiliations:** CAS Key Laboratory of Pathogenic Microbiology & Immunology, Institute of Microbiology, Chinese Academy of Sciences, Beijing, 100101 PR China; Collaborative Innovation Center for Diagnosis and Treatment of Infectious Diseases, The First Affiliated Hospital, College of Medicine, Zhejiang University, Hangzhou, 310006 China; Department of Microbiology and Immunology, Institute of Biomedicine, University of Gothenburg, Box 435, 405 30 Göteborg, Sweden; International Centre for Diarrhoeal Disease Research, Bangladesh, GPO Box 128, Dhaka, 1000 Bangladesh; Clinical Diagnostic Center, 306nd Hospital of the People’s Liberation Army, Beijing, 100101 PR China; Department of Microbiology, Tumor and Cell Biology, Karolinska Institutet, Box 280, 171 77 Stockholm, Sweden

**Keywords:** ETEC, Virulence factors, Antibiotic resistance

## Abstract

**Background:**

Enterotoxigenic *Escherichia coli* (ETEC) is an important pathogen that causes childhood and travelers’ diarrhea. Here, we present the draft genomes of four ETEC isolates recovered from stool specimens of patients with diarrhea in Beijing, China and Dhaka, Bangladesh, respectively.

**Results:**

We obtained the draft genomes of ETEC strains CE516 and CE549 isolated in China, and E1777 and E2265 isolated in Bangladesh with a length of 5.1 Mbp, 4.9 Mbp, 5.1 Mbp, and 5.0 Mbp, respectively. Phylogenetic analysis indicated that the four strains grouped with the classical *Escherichia coli* phylogenetic groups A and B1 and three of them including a multi drug-resistant Chinese isolate (CE549) belonged to two major ETEC lineages distributed globally. The heat stable toxin (ST) structural gene (*estA*) was present in all strains except in strain CE516, and the heat labile toxin (LT) operon (*eltAB*) was present in all four genomes. Moreover, different resistance gene profiles were found between the ETEC strains.

**Conclusions:**

The draft genomes of the two isolates CE516 and CE549 represent the first genomes of ETEC reported from China. Though we revealed that ETEC is uncommon in Beijing, China, however, when it does occur, multi-drug resistance and ESBL positive isolates might pose a specific public health risk. Furthermore, this study advances our understanding of prevalence and antibiotic resistance of ETEC in China and adds to the number of sequenced strains from Bangladesh.

## Background

ETEC infections are an important cause of childhood diarrhea resulting in significant morbidity and mortality, primarily among children aged <5 years living in developing countries [1] as well as travelers visiting these countries [2]. ETEC is characterized by the presence of the heat-labile toxin (LT) and/or the heat-stable toxin (ST), both of which are plasmid encoded [3]. The presence of virulence factors such as enterotoxins and colonization factors differentiate ETEC from other categories of diarrheagenic *E. coli.* [4]. Colonization factors (CFs) enable ETEC bacteria to adhere to the intestinal epithelium [5]. At present more than 25 different CFs have been identified [5]. In addition to the CFs, other putative factors involved in ETEC pathogenesis were also identified, such as EtpA and EatA. EtpA can act as a bridge between the bacterial flagella and host epithelial cells [6] and EatA is a protein of the serine protease autotransporters of the *Enterobacteriaceae* (SPATE) family [7].

For a long time *E. coli* H10407 and E24377A were the only two ETEC strains infecting humans that have their genomes completely sequenced together with a draft genome of ETEC strain B7A [8,9]. Recently whole genome sequences of additional draft genomes were published [10]. A comprehensive analysis of 362 ETEC genomes from strains isolated globally over three decades identified that ETEC distribute into several conserved monophyletic lineages that have distrubuted globally [11] . In this study we analysed four additional ETEC strains with the aim to compare additional ETEC isolated in China and Bangladesh with the global collection and to better understand the dissemination of the pathogen. We also included two additional Bangladeshi strains to increase the number of sequenced genomes from Bangladeshi ETEC strains.

## Methods

### Strain selection

To assess the frequency of ETEC in Beijing, China, we investigated patients presenting with acute watery diarrhea at four hospitals between 2010 and 2011. This research was approved by the Research Ethics Committee of the Institute of Microbiology, Chinese Academy of Sciences. ETEC isolates were recovered after streaking diarrheal samples on to MacConkey agar followed by PCR confirmation for ETEC-specific enterotoxins [12]. In total, 880 cases were enrolled and tested for ETEC but ETEC was only recovered from three cases (0.3%). The two ETEC isolates CE516 and CE549 from China were recovered from stool of patients that tested negative for *Vibrio cholerae, Shigella* spp and *Salmonella* spp. CE549 expressed the heat-labile enterotoxin (LT) and the human heat-stabile enterotoxin (STh) in combination with CFs CS2, CS3 and CS21; CE516 expressed LT and CS6, CS8. Antimicrobial susceptibility was determined using the VITEK 2 Gram Negative Susceptibility Test Cards AST-GN04 and AST-GN 13 (Biomerieux, Marcy l’Etoile France). CE549 was resistant to 14 of the 22 antibiotics tested (cefuroxime axetil, sulfamethoxazole, ampicillin, tobramycin, ceftriaxone, aztreonam, piperacillin, cefuroxime, cefazolin, ceftazidime, cefepime, levofloxacin, gentamicin, ciprofloxacin, and extended spectrum beta lacatamase (ESBL) positive), while CE516 showed sensitive to all 22 antibiotics and was ESBL negative.

The two ETEC isolates E1777 and E2265 were collected from adult Bangladeshi patients that sought medical attention for severe diarrhea in hospital facilities in April 2005 and March 2006 during the bi-annual ETEC epidemic peaks in Dhaka, Bangladesh [13]. Stool samples were confirmed to be negative for *Vibrio cholerae, Shigella ssp* and *Salmonella ssp.* MacConkey agar plates were used for identification of lactose fermenting *E. coli* like colonies selection followed by PCR confirmation for ETEC [12]. The strains were further characterized by immunodiagnostic methods for toxins and colonization factors [12]. Both isolates expressed the common virulence factor combination of the enterotoxins heat labile toxin LT and heat stable toxin STh and the CFs CS5 and CS6.

### Genome sequencing, assembly and annotation

DNA was extracted from bacterial cells cultured in Luria broth (LB) medium using the DNA Tissue and Blood kit (Qiagen, Duesseldorf, Germany). Genome sequencing work was carried out at the Microbial Genome Research Center, Institute of Microbiology, Chinese Academy of Sciences, Beijing. The genome sequences of each ETEC isolate were generated using paired-end libraries with 350 ~ 400 bp inserts on an Illumina GAIIX (Illumina, San Diego, CA, USA). The detailed methods for genome assembly were described in another paper [14]. Genome sequences were annotated by using Subsystem Technology (RAST) [15]. The functions of predicted protein-coding genes were then annotated through comparisons with the databases of NCBI-NR, and COG. To search the antibiotic resistance genes, the protein-coding sequences were aligned against Antibiotic Resistance Database (ARDB) [16], using similarity thresholds as recommended in ARDB.

### Multiple locus sequence typing (MLST)

We used MLST system including the following seven housekeeping genes: *adk, fumC, gyrB, icd, mdh, purA, and recA* [17]*,* which were extracted from draft genome sequences and were compared to allele profiles in the MLST database (http://mlst.warwick.ac.uk/mlst/dbs/Ecoli/documents/primersColi_html).

### Comparative genomics

For comparative genomic analysis, genome sequences of 13 previously reported isolates including *Escherichia coli* B7A (GenBank accession number NZ_AAJT02000001.1), E24377A (NC_009786.1), H10407 (NC_017633.1), IAI39 (NC_011750.1), O127 H6 E2348 69 (NC_011601.1), O157 H7 EC4115 (NC_011350.1), O157 H7 EDL933 (NC_002655.2), O157 H7 TW14359 (NC_013008.1), O157 H7 Sakai (NC_002127.1), SMS 3 5 (NC_010485.1), TW10598 (NZ_AELA01000001.1), TW10722 (NZ_AELB01000001.1), and TW10828 (NZ_AELC01000001.1) were downloaded from the NCBI website (Table 1). Multiple sequence alignments of *Escherichia coli* genomes were performed with Mugsy [18]. The trees were constructed based on core SNPs (single nucleotide polymorphisms) from whole genome alignment by using the maximum-likelihood method in Phylogeny Inference Package (http://evolution.genetics.washington.edu/phylip.html). The map of ORF comparisons among *E. coli* genomes was constructed using Circos [19].

**Table 1:** **Reference strains used for this study**

**Strain**	**GenBank BioSample**	**Accession number**	**Collection date**	**Isolation source**	**Genome size (bp)**	**GC content**
B7A	SAMN02435852	NZ_AAJT02000001.1	\	\	5,300,242	50.7%
E24377A	SAMN02604038	NC_009786.1	\	\	5,249,288	50.6%
H10407	SAMEA2272237	NC_017633.1	prior to 1973	\	5,325,888	50.7%
IAI39	SAMEA3138234	NC_011750.1	\	\	5,132,068	50.6%
O127 H6 E2348/69	SAMEA1705959	NC_011601.1	1969	\	5,069,678	50.5%
O157 H7 EC4115	SAMN02603441	NC_011350.1	\	\	5,704,171	50.4%
O157 H7 EDL933	SAMN02604092	NC_002655.2	\	\	5,620,522	50.4%
O157 H7 TW14359	SAMN02604255	NC_013008.1	\	\	5,622,737	50.5%
O157 H7 Sakai	SAMN01911278	NC_002127.1	1996	Human feces	5,594,477	50.5%
SMS-3-5	SAMN02604066	NC_010485.1	\	\	5,215,377	50.5%
TW10598	SAMN02436015	NZ_AELA01000001.1	\	\	5,243,318	50.6%
TW10722	SAMN02435971	NZ_AELB01000001.1	\	\	5,689,893	50.5%
TW10828	SAMN02435898	NZ_AELC01000001.1	\	\	5,280,267	50.6%

## Quality assurance

The genomic DNA was isolated from pure bacterial isolate and was further confirmed with 16S rRNA gene sequencing. Bioinformatic assessment of potential contamination of the genomic library by allochthonous microorganisms was done using PGAAP and RAST annotation system.

## Initial findings

### Genome characteristics

Through genome assembly, we obtained 99 scaffolds of 5,068,634 bp for CE516, 137 scaffolds of 4,859,890 bp for CE549, 150 scaffolds of 5,117,746 bp for E1777, and 142 scaffolds of 4,946,932 bp for E2265 (Table 2). RAST annotation of the whole genome indicated the presence of 611, 590, 605, and 605 SEED subsystems in CE516, CE549, E1777, and E2265, respectively. Table 3 shows the comparison of genomic features of the four sequenced ETEC genomes.

**Table 2:** **Genomic characteristics of the 4 ETEC genomes**

**Sample name**	**Country**	**MLST**	**Colonization factors**	**ST**	**LT**	**Read length (bp)**	**Genome coverage**	**GC content**	**Scaffold number**
CE516	China	1490	CS6, CS8	-	+	101	300x	50.5%	99
CE549	China	4	CS2, CS3, CS21	+	+	101	300x	50.6%	137
E1777	Bangladeshi	443	CS5, CS6	+	+	101	200x	50.4%	150
E2265	Bangladeshi	443	CS5, CS6	+	+	101	200x	50.3%	142

**Table 3:** **Comparisons of subsystem features among the 4 ETEC genomes**

**Subsystem features**	**Number of CDS present in ETEC strains**			
	**CE516**	**CE549**	**E1777**	**E2265**
Amino Acids and Derivatives	400	391	392	395
Carbohydrates	781	756	752	754
Cell Division and Cell Cycle	39	40	38	37
Cell Wall and Capsule	267	273	272	273
Cofactors, Vitamins, Prosthetic Groups, Pigments	285	285	287	284
DNA Metabolism	129	147	153	134
Dormancy and Sporulation	4	5	5	5
Fatty Acids, Lipids, and Isoprenoids	142	131	132	131
Iron acquisition and metabolism	22	22	22	22
Membrane Transport	291	190	268	270
Metabolism of Aromatic Compounds	44	5	30	30
Miscellaneous	67	63	66	64
Motility and Chemotaxis	80	130	80	80
Nitrogen Metabolism	77	75	77	77
Nucleosides and Nucleotides	146	150	147	144
Phages, Prophages, Transposable elements, Plasmids	130	32	160	146
Phosphorus Metabolism	53	53	53	53
Photosynthesis	0	0	0	0
Potassium metabolism	29	29	28	30
Protein Metabolism	299	290	298	300
Regulation and Cell signaling	160	156	160	163
Respiration	192	190	194	192
RNA Metabolism	248	251	250	250
Secondary Metabolism	27	26	26	26
Stress Response	184	181	186	184
Sulfur Metabolism	59	54	56	56
Virulence, Disease and Defense	109	108	110	130

### Phylogenetic analysis

**Figure 1:**
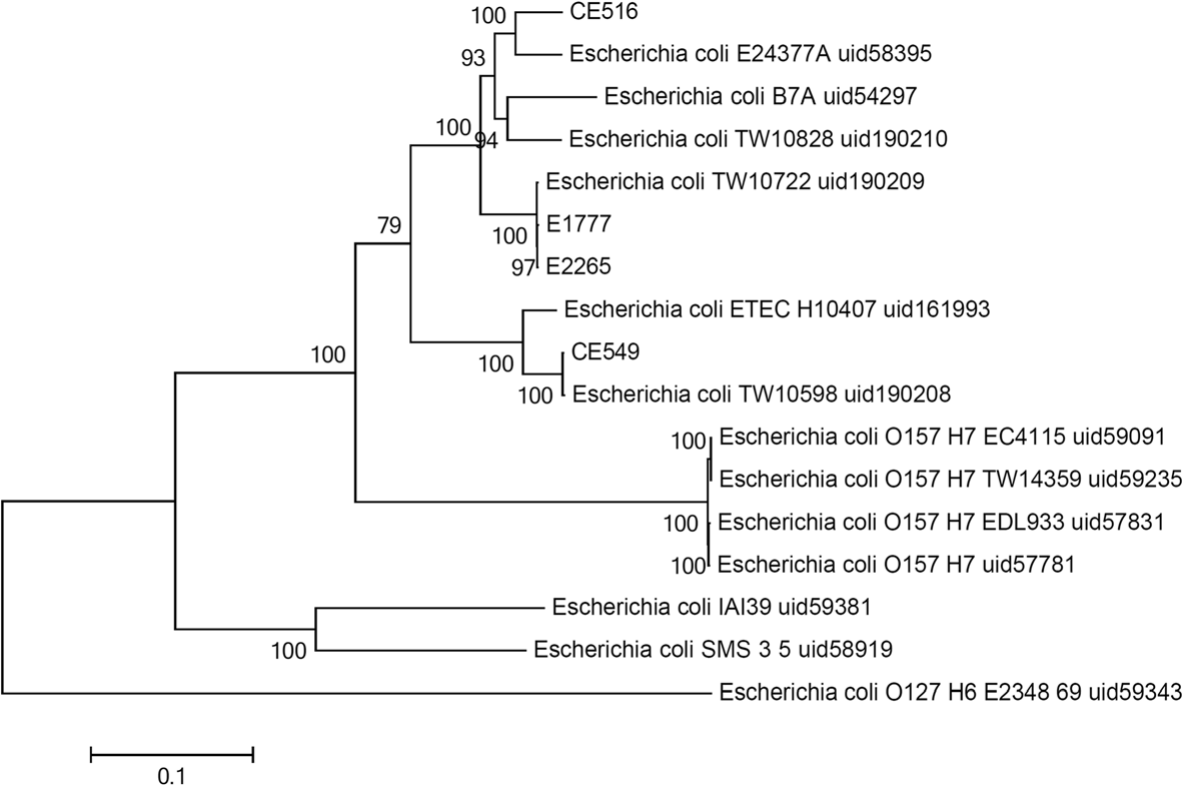
**Phylogenetic relationships of** ***E. coli*** **strains based on SNPs from whole genome sequences.** The trees were constructed by the maximum-likelihood method. Scale bar indicates nucleotides substitutions per site.

A maximum-likelihood tree of the sequenced 4 genomes and 13 publicly available *Escherichia coli* complete genomes which represent the classical phylogenetic groups (A, B1, B2, D, and E) were created based on core SNPs from whole genome alignment (Figure 1). The sequenced strains in this study grouped with the classical *Escherichia coli* phylogenetic groups A and B1. Specifically, strains CE549, H10407 and TW10598 which belong to group A were grouped together, while other sequenced strains which belong to group B1 as well as the previously sequenced strains formed a clade. Strains CE549 and TW10598 are closely related to each other, while strains E1777 and E2265 are closely related to each other. MLST analysis was used to compare the strains to a global collection of ETEC [11]. Three strains were found to belong to the major lineages described in ETEC [11]. Strains E1777 and E2265 belong to the global lineage L5 which express LT STh CS5 + CS5, while strain CE156, the multi drug-resistant isolate belongs to the conserved ETEC lineage L2 that is distributed globally [11]. The Chinese strain CS516 belonged to a MLST type previously identified in Bangladeshi and Egyptian ETEC strains [11].

### Genomic variants among ETEC strains

**Figure 2:**
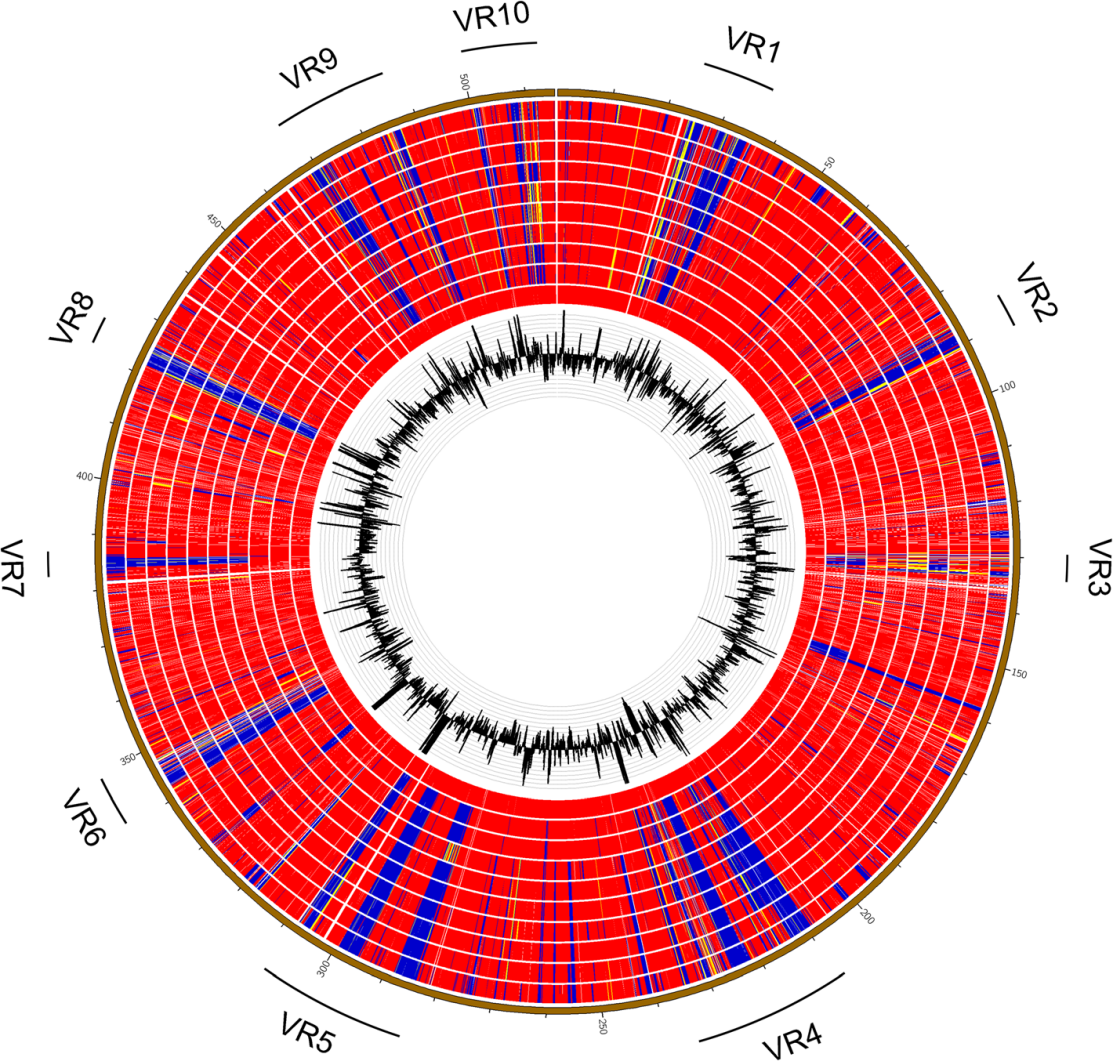
**ORF comparisons of** ***E. coli*** **genomes.** Proteins from the 4 genomes and 6 references within groups A and B1 were aligned using H10407 as a reference. Track shows a plot of G + C contents. Circles from inside to outside are the BLASTP percent identities of H10407 against ORFs of H10407, TW10598, CE549, TW10722, E1777, E2265, E24377A, CE516, TW10828, B7A. Red is 90–100% identity, yellow is 60–89% identity, blue is 0–59% identity.

We compared proteins from the 4 draft genomes and 6 references within groups A and B1 with that from H10407 using BLASTP and revealed many large variable regions (VR1 to VR10) (Figure 2). Among these VRs, VR3 and VR10 (regions of 5,072 to 5,121 kb) were predicted to be prophage loci which were highly variable among all strains. Interestingly, all strains within group B1 lack VR7 gene cluster encoding general secretory pathway associated genes. In addition, region 2,405 to 2,414 kb adjacent to VR4, which encoded ribitol metabolism related genes, was presented within group A but not detected within group B1.

### Virulence factors

The strains were analyzed for presence of known ETEC virulence factors. Strains E1777, E2265, and CE549 contained both LT and ST genes (Table 4). The ST structural gene (*estA*) was present in all strains except in strain CE516, while the LT structural gene (*eltA*) was present in all four genomes. In addition, genes *clyA* (cytolysin), *eatA* (serine protease autotransporter), and *ecpA* (pilus subunit) were also present in all of the 4 ETEC strains, but genes *leoA* (accessory protein for LT secretion), *tibA* (autotransporter), and *tia* (surface protein) were absent in all genomes. Only CE549 contained the complete ~14 kb operon encoding longus known as a type IV pilus [20]. The *etpA* gene, which mediates adhesion between ETEC flagella and host cells [6], was present only in CE549 but absent in other strains. These specific virulence factors present in CE549 may increase its virulence in humans, but their functional effects remain to be further determined.

**Table 4:** **Virulence factors present or absent in the 4 ETEC genomes**

**Virulence factor**	**CE516**	**CE549**	**E1777**	**E2265**
clyA	1	1	1	1
eatA	1	1	1	1
ecpA	1	1	1	1
eltA	1	1	1	1
estA	0	1	1	1
etpA	0	1	0	0
fimH	1	1	1	1
leoA	0	0	0	0
lngA	0	1	0	0
tia	0	0	0	0
tibA	0	0	0	0

“1” and “0” denotes the presence and absence of the corresponding virulence factors.

### Antibiotic resistance genes

We compared all the protein-coding genes from the 4 ETEC strains with known antibiotic resistance genes [16] and found many kinds of antibiotic resistance genes, such as macrolide, tetracycline, fosmidomycin and polymyxin resistance genes (Table 5), most of which were annotated as Multidrug resistance efflux pump. Interestingly, strain CE549 has two tetracycline resistance genes that were not identified in the other 3 isolates. In addition, different resistance genes profiles were found between ETEC strains from different countries. For instance, the resistant type EmrE was only identified in the two strains isolated from China.

**Table 5:** **Putative antibiotic resistance genes in the 4 ETEC strains determined using the antibiotic resistance genes database**

**Resistance type**	**Description**	**Resistance profile**	**CE516**	**CE549**	**E1777**	**E2265**
acrA	Multidrug resistance efflux pump.	aminoglycoside, glycylcycline, beta_lactam, macrolide, acriflavin	*	*	*	*
acrB			*, *	*, *	*, *, *	*, *, *
arnA	The modified arabinose is attached to lipid A and is required for resistance to polymyxin and cationic antimicrobial peptides.	polymyxin	*	*	*	*
bacA	Undecaprenyl pyrophosphate phosphatase, which consists in the sequestration of Undecaprenyl pyrophosphate.	bacitracin	*	*	*	*
bcr			*	*	*	*
bl1_ec	Class C beta-lactamase.	cephalosporin	*	*	*	*
emrD	Multidrug resistance efflux pump.		*	*	*	*
emrE		aminoglycoside	*, *, *	*		
ksgA	Its inactivation leads to kasugamycin resistance.	kasugamycin	*	*	*	*
macB	Macrolide-specific efflux system.	macrolide	*	*	*	*
mdfA			*	*	*	*
mdtE	Multidrug resistance efflux pump.	doxorubicin, erythromycin	*	*	*	*
mdtF			*	*	*	*
mdtG	Multidrug resistance efflux pump.	deoxycholate, fosfomycin	*	*	*	*
mdtH			*	*	*, *	*, *
mdtK		enoxacin, norfloxacin	*	*	*	*
mdtL		chloramphenicol	*	*	*	*
mdtM		chloramphenicol, acriflavine, norfloxacin	*	*	*	*
mdtN	Multidrug resistance efflux pump.	t_chloride, acriflavine, puromycin	*	*	*	*
mdtO			*	*	*	*
mdtP			*	*	*	*
rosB	Efflux pump/potassium antiporter system. RosB: Potassium antiporter.	fosmidomycin	*	*	*	*
tetC	Major facilitator superfamily transporter, tetracycline efflux pump.	tetracycline		*, *		
tolC	Multidrug resistance efflux pump.	aminoglycoside, glycylcycline, beta_lactam, macrolide, acriflavin	*	*	*	*

“*” means one homolog of the antibiotic resistance gene is found.

## Future directions

This study analyzed the prevalence of ETEC in Beijing, China and it was found that ETEC is not common. However the results reveal for the first time to our knowledge that a strain that belong to the globally distributed ETEC lineage L2 is multi resistant. This might have important implications for transmission of multi resistant ETEC strains as well as treatment of ETEC diarrhea and needs to be further addressed. The Chinese genomes presented here together with the two novel Bangladeshi ETEC genomes, will be valuable for future comparative genomic analysis of ETEC and will aid in molecular characterization of this important diarrheal pathogen.

## Availability of supporting data

The genome sequences of ETEC strains CE516, CE549, E1777 and E2265 reported in this paper have been deposited in the GenBank under accession numbers JTGM00000000, JTGK00000000, JTHI00000000 and JUBB00000000, respectively.
